# Vaccine-induced antibodies can limit *Salmonella* infection in the absence of complement or macrophages

**DOI:** 10.1128/mbio.02846-25

**Published:** 2026-02-25

**Authors:** Marisol Perez-Toledo, Kubra Aksu-Istil, Edith Marcial-Juarez, Ruby R. Persaud, Areej Alshayea, Sian E. Jossi, Agostina Carestia, Fien von Meijenfeld, Marina Botto, Leo C. James, Bas Surewaard, Zaheer Afzal, Adrian M. Shields, William G. Horsnell, Constantino Lopez-Macias, Ian R. Henderson, OptiVaNTS consortium, Galit Alter, Craig N. Jenne, Adam F. Cunningham

**Affiliations:** 1 Institute of Immunology and Immunotherapy, University of Birmingham, Birmingham, United Kingdom; 2 Department of Clinical Laboratory Sciences, College of Applied Medical Sciences, King Saud University 37850, Riyadh, Saudi Arabia; 3 Department of Microbiology, Immunology and Infectious Diseases, Cumming School of Medicine, University of Calgary 2129, Calgary, Canada; 4 University Medical Center Groningen, Groningen, the Netherlands; 5 Department of Immunology and Inflammation, Centre for Inflammatory Disease, Imperial College London 4615, London, United Kingdom; 6 MRC Laboratory of Molecular Biology 47694, Cambridge, United Kingdom; 7 Clinical Immunology Service, Institute of Immunology and Immunotherapy, University of Birmingham 152871, Birmingham, United Kingdom; 8 Division of Immunology, Institute of Infectious Disease and Molecular Medicine, University of Cape Town 71985, Cape Town, South Africa; 9 Medical Research Council Centre for Medical Mycology, University of Exeter 3286, Exeter, United Kingdom; 10 Medical Research Unit in Immunochemistry, Specialty Hospital, National Medical Center Siglo XXI, Mexican Institute of Social Security, Mexico City, Mexico; 11 Institute for Molecular Bioscience, University of Queensland 85088, Brisbane, Australia; GSK Vaccines, Siena, Italy

**Keywords:** *Salmonella*, antibodies, complement, intravital imaging, macrophages, neutrophils

## Abstract

Antibodies against *Salmonella* Typhimurium (STm) can provide protection against infection. Understanding how antibodies, complement, and leukocytes interact is essential and can help advance vaccine development. To evaluate the *in vivo* role of STm-specific antibodies, mice were immunized with an outer membrane vesicle (OMV) vaccine and subsequently challenged with STm. Immunohistology and intravital microscopy revealed that OMV-induced antibodies promoted STm uptake by macrophages in the spleen and liver, whereas bacteria were only infrequently associated with neutrophils. Depletion of monocytic cells using clodronate liposomes demonstrated that these cells help prevent antigen dissemination. Immunization and challenge experiments in mice deficient in C1q, C3, C4, or C5 showed that OMV immunization conferred protection in all groups except C3-deficient mice. Mice deficient in C3 failed to develop robust germinal center and plasma cell responses following OMV immunization. Nevertheless, C3-deficient mice that received immune sera by adoptive transfer prior to infection had significantly reduced bacterial burdens compared to mice receiving non-immune control sera. *In vitro* studies using human sera and THP-1 cells confirmed that antibodies alone are sufficient to promote bacterial capture by macrophages. Complement—particularly C1q and C3, but not C5—further enhanced bacterial uptake. Overall, under the conditions tested, antibodies are sufficient to reduce STm burdens *in vivo* and facilitate bacterial uptake *in vitro*, with these processes supported by complement and macrophages.

**IMPORTANCE:**

Bacterial infections remain a significant global challenge, further complicated by the growing issue of antimicrobial resistance (AMR). In this context, vaccination provides a cost-effective means of preventing infections and combating the emergence of AMR strains. Antibodies are key mediators of vaccine-induced protection. However, their mechanisms of action *in vivo* are not fully understood. In this study, we demonstrate how antibodies enhance the capture of *Salmonella* by macrophages. Although these cells may be dispensable for controlling bacterial numbers, they are crucial for limiting the spread of bacterial antigens. Additionally, we find that the classical complement cascade is not necessary for bacterial clearance but does facilitate bacterial capture by macrophages. Overall, our findings reveal that multiple antibody-mediated pathways operate *in vivo*, exhibiting some redundancy in their combined efforts to control infection, underscoring the importance of antibody functions in limiting bacterial spread.

## INTRODUCTION

Infectious diseases remain a leading threat to human health. In 2019, approximately 13.7 million deaths were attributed to 33 bacterial pathogens, making bacterial infections the second-leading cause of death worldwide. Infections caused by *Salmonella*, including *Salmonella* Typhi and non-typhoidal *Salmonella* (NTS), were among the top 14 causes of infection-related deaths worldwide (1, 2). NTS infections typically cause self-limiting diarrhea; however, invasive NTS infections are more severe and are often associated with high mortality rates in at-risk groups, such as infants in sub-Saharan Africa and individuals with HIV (3). Currently, there are no licensed vaccines against iNTS, although vaccine candidates are being assessed in clinical trials, including those based on outer membrane vesicles (OMV) (4, 5). Understanding how immune responses to these types of vaccines work may support efforts to develop vaccines for *Salmonella* and other invasive gram-negative bacteria.

Antibodies play a crucial role in the protection provided by many vaccines and can also be correlates of protection. For *Salmonella*, targeting surface antigens by antibodies, such as the lipopolysaccharide O-antigen or porins, is sufficient to reduce bacterial infection in animal models (6–8). Indeed, the two leading vaccine candidates against iNTS both contain O-antigen. Furthermore, antibodies against *Salmonella* Typhimurium (STm) O-antigen strongly correlate with protection against iNTS infections in humans (9). Mechanistically, antibodies, phagocytic cells, and complement can aid in bacterial killing *in vitro* (10). However, *in vitro*, opsonophagocytic uptake of *Salmonella* by leukocytes can occur more rapidly than complement-mediated, cell-independent killing (11). This suggests that when both cell-dependent and cell-independent mechanisms of bacterial control are present, the cell-dependent pathway may be the primary mechanism by which antibodies exert their function. Nevertheless, antibodies and complement-derived opsonins can also engage Fc gamma (Fcγ) receptors and complement receptors on immune cells, thereby enhancing phagocytosis (12). Thus, both complement and cells could collaborate with antibodies to optimize bacterial control. It remains to be determined whether the cooperative function of anti-*Salmonella* antibodies with phagocytic cells and complement also occurs *in vivo*.

In this work, we explored the requirements of selective factors associated with vaccination-induced, antibody-mediated protection *in vivo*. To do this, we immunized and challenged mice with an OMV-based vaccine derived from STm and then challenged them with live bacteria. In this model, antibodies are essential for mediating protection (13). By employing a combination of intravital and other imaging approaches, we find that the critical factor in the protection provided by immunization is the presence of antibodies, and multiple pathways are active, enabling these functions primarily to control bacterial numbers and localization throughout the host.

## RESULTS

### STm preferentially associates with macrophages in the spleen and liver, regardless of vaccination

**Fig 1:**
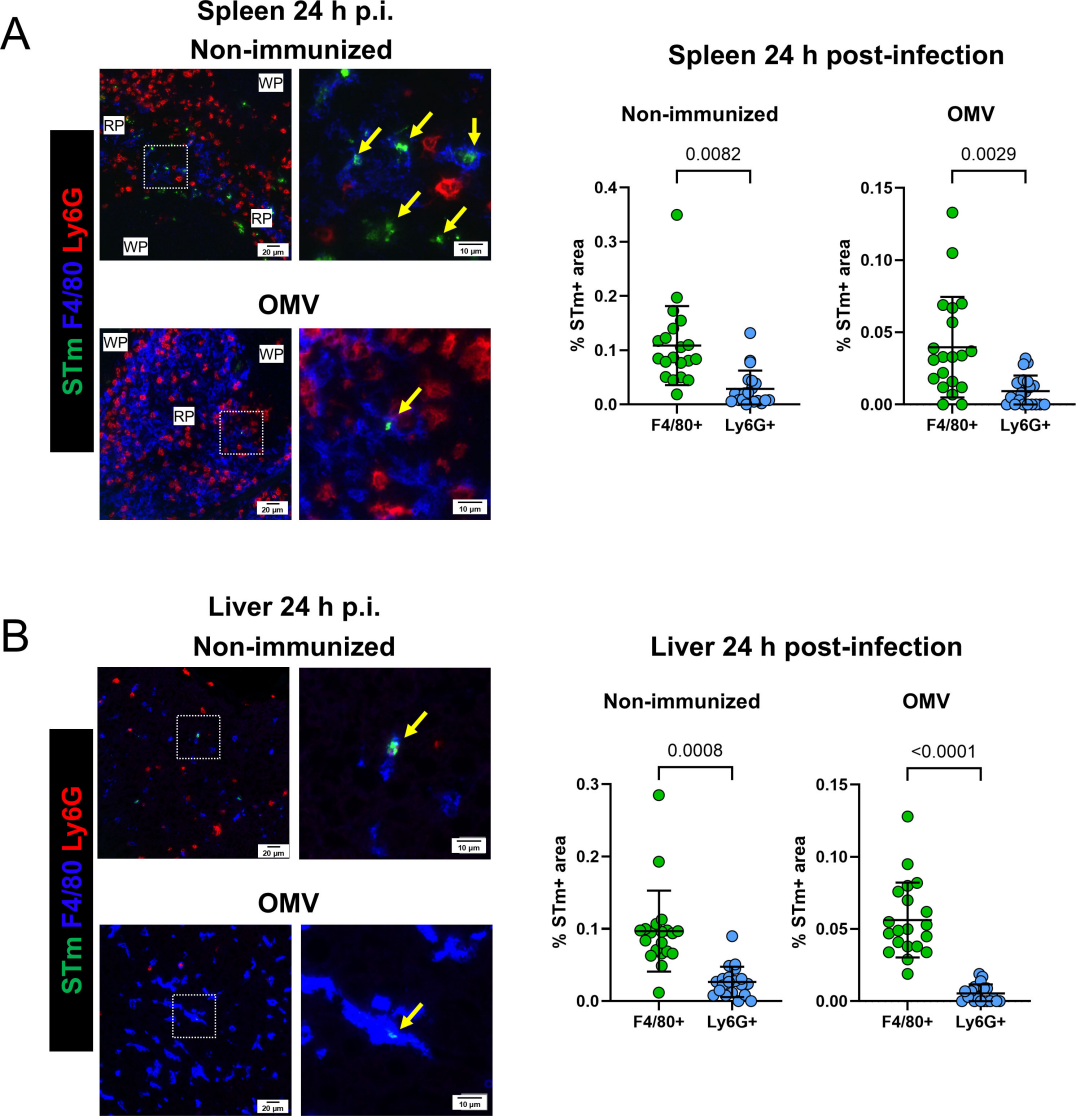
STm preferentially associates with macrophages in the spleen and liver, regardless of immunization. Non-immunized and OMV-immunized wild-type (WT) mice (*n* = 4 per group) were challenged with STm SL3261 for 24 h. Spleen (**A**) and liver (**B**) microsections were stained for F4/80 (blue), Ly6G (red), and STm (green). Arrows indicate positive bacterial staining. Secondary scale bars were added to the original images to ensure consistency throughout the figure. The graphs on the right show the percentage of STm-positive pixel area associated with F4/80^+^ or Ly6G^+^ cells. Each dot represents a random field of view, with five fields of view assessed per tissue section from four mice per group, for a total of 20 assessments per group. Data representative of three experiments. Data shown as mean ± SD. Two-tailed Mann-Whitney *U* test.

Different mouse strains immunized with OMV and challenged with STm of varying virulence have lower bacterial burdens than non-immunized controls (13, 14). This is also observed when C57BL/6 mice are infected with virulent STm LT2 (Fig. S1). To evaluate which leukocyte population associates with STm following immunization and challenge, spleen and liver tissue sections from mice that were infected for 24 h were stained for STm, macrophages (F4/80^+^), and neutrophils (Ly6G^+^). In the spleen, both non-immunized and immunized mice showed that STm was restricted to the red pulp, with bacteria primarily associated with F4/80^+^ cells (Fig. 1A). Similarly, in the liver, STm was found to be mainly associated with F4/80^+^ cells (Fig. 1B). Therefore, STm associates with macrophages in the spleen and liver, regardless of immunization status.

### Immunization with OMVs promotes bacterial capture in the first minutes of infection

**Fig 2:**
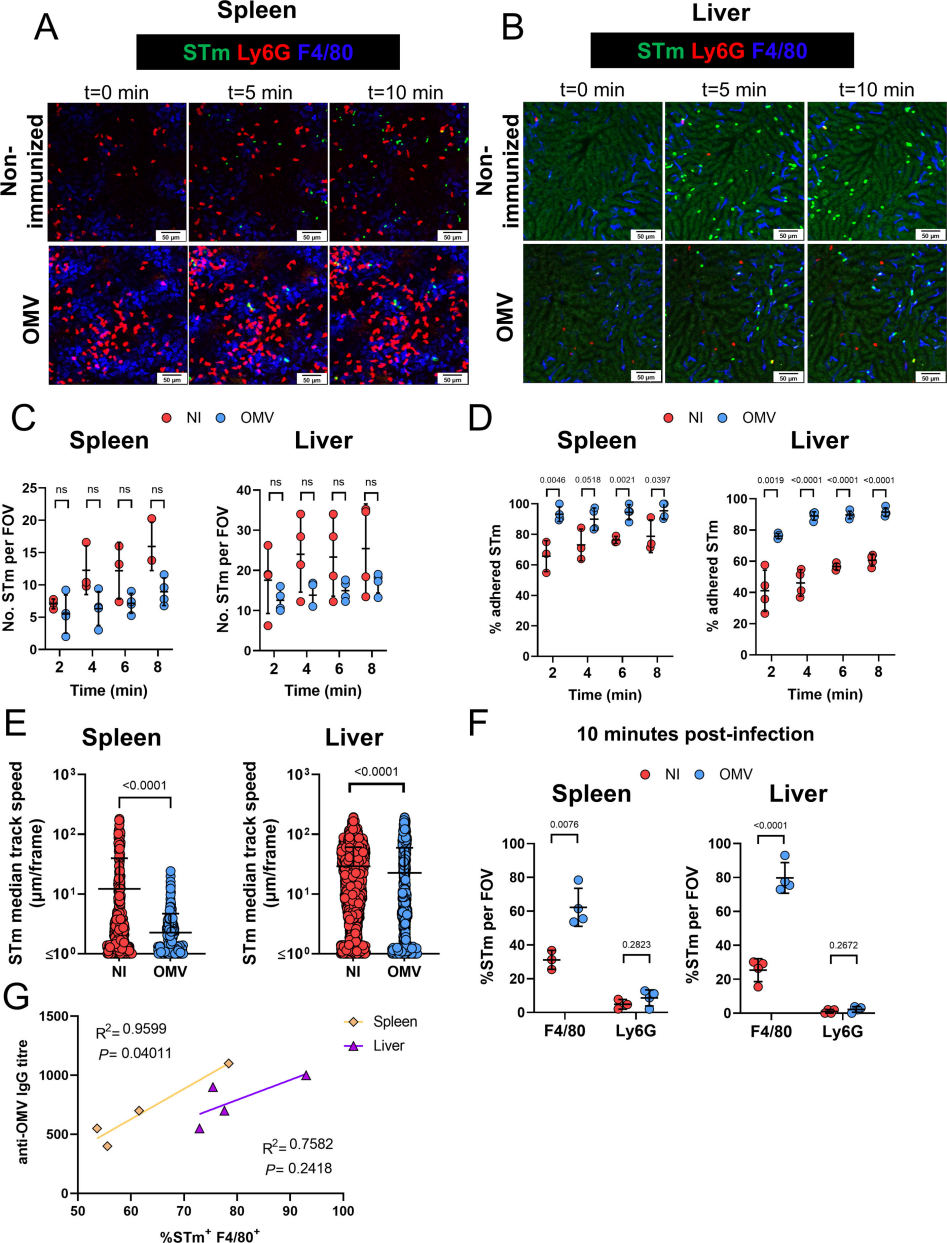
Immunization with OMVs enhances the capture of STm by F4/80^+^ cells in the spleen and liver. Non-immunized and OMV-immunized wild-type (WT) mice (*n* = 3–4 per group) were challenged i.v. with 10^7^ CFU of GFP-expressing STm, and videos were recorded for 10 min post-infection. Representative images of spleen (**A**) and liver (**B**) at times (T) 0, 5, and 10 min after infection. Green = STm, red = Ly6G, and blue = F4/80. Secondary scale bars were added to the original images to ensure consistency throughout the figure. (**C**) Number of STm per field of view (FOV) at the indicated time points post-challenge. (**D**) Frequency of adhered STm per FOV at the indicated time points post-challenge. Two-tailed unpaired *t*-test. (**E**) Median track speed of STm in spleens and livers of non-immunized (NI) and OMV-immunized mice. Each point represents an individual track assessed from three different fields of view, combined from 3 to 4 mice per group. (**F**) Frequency of STm associated with F4/80^+^ cells or Ly6G^+^ cells after 10 min of STm challenge. (**G**) Spearman’s correlation of anti-OMV IgG titers and frequency of STm associated with F4/80^+^ cells in the spleen and liver. In **C**, **D**, **F**, and **G**, each point represents values from a single mouse. The data shown are from two combined experiments. Data shown as mean ± SD. Two-tailed Mann-Whitney *U* test. ns = non-significant.

Our results at 24 h post-challenge suggested that F4/80^+^ cells were the primary cell type associated with STm after infection, with minimal bacterial association with neutrophils. However, this could be the result of more efficient bacterial clearance by neutrophils, which would not be detected in our analysis at 24 h post-challenge. To understand the earliest interactions between STm, macrophages, and neutrophils, intravital microscopy experiments were conducted, in which non-immunized and immunized mice were challenged with a GFP-expressing STm strain. In both non-immunized and immunized mice, bacteria were detected throughout the spleen and liver 10 min post-infection. Bacteria could be observed as early as 30 s after challenge (Fig. 2A through C and Videos S1 through S4). In immunized mice, the proportion of adhered bacteria over time was higher compared to non-immunized controls (Fig. 2D). Moreover, in immunized mice, bacteria showed a lower median speed, suggesting that more bacteria were adhered (Fig. 2E). Additional assessments revealed that most bacteria associated with F4/80^+^ cells rather than Ly6G^+^ cells after infection for 10 min (Fig. 2F), but this association with F4/80^+^ cells was most significant in vaccinated mice (Fig. 2F). The association of STm with F4/80^+^ cells showed a positive correlation with the anti-OMV IgG titers (Fig. 2G). The association of bacteria with F4/80^+^ cells was also observed at an intermediate time point (6 h; Fig. S2 and Videos S5 and S6). Thus, OMV immunization promotes bacterial association with F4/80+ cells, which correlates with anti-STm antibody titers.

### Bacterial numbers are reduced after challenge of OMV-immunized, monocytic cell-depleted mice

**Fig 3:**
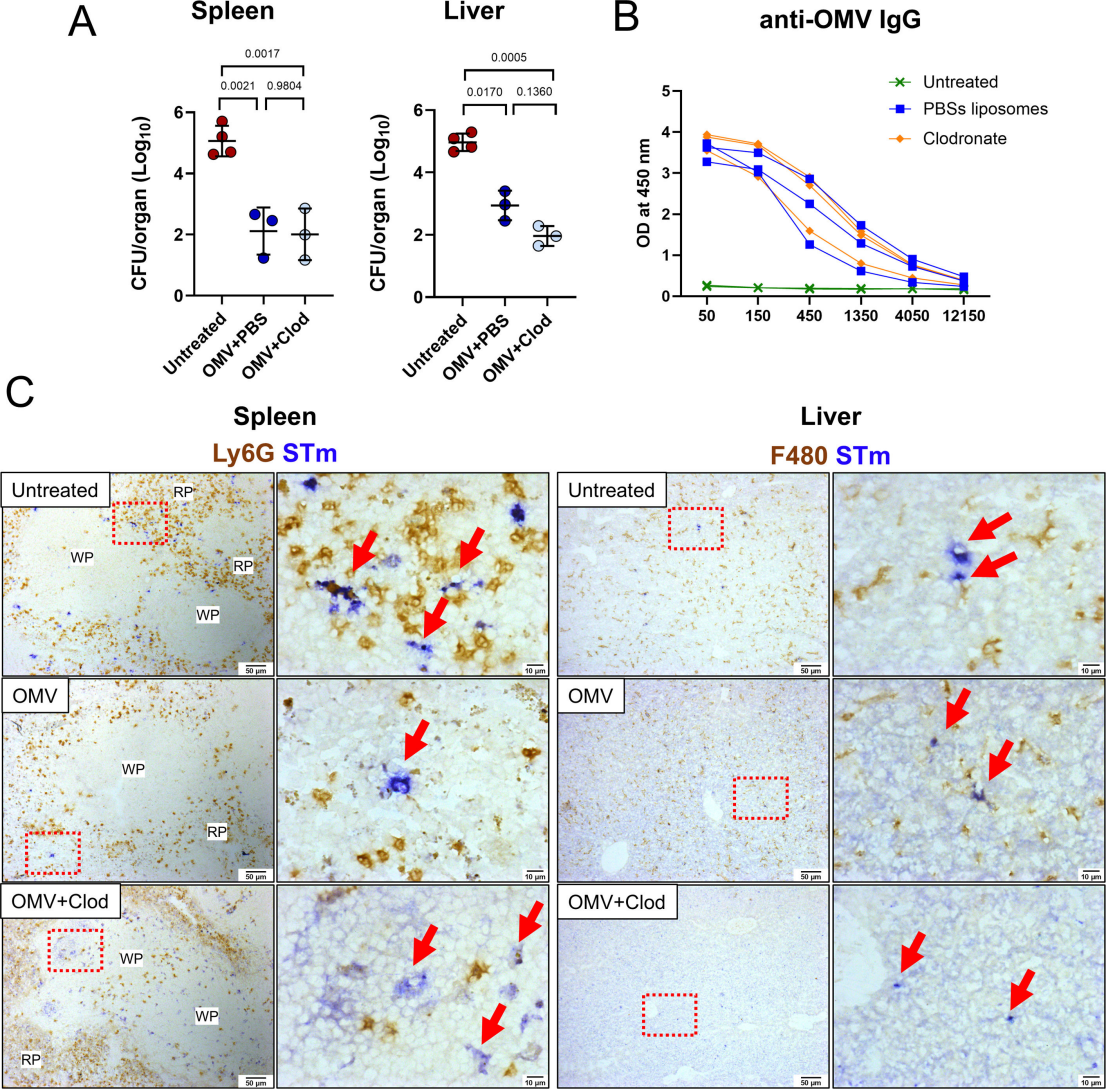
Bacterial numbers are reduced after the challenge of OMV-immunized, monocytic cell-depleted mice. Wild-type (WT) mice were vaccinated with OMVs and, 12 days later, treated with PBS liposomes (OMV + PBS) or clodronate liposomes (OMV + clod). Untreated mice did not receive OMV or liposomes. All three groups were challenged with STm for 24 h. (**A**) Spleen and liver bacterial burdens in untreated (*n* = 4), OMV + PBS (*n* = 3), and OMV + Clod (*n* = 3) mice. Each point represents values from a single mouse. (**B**) Anti-OMV IgG in sera from mice in panel **A**; each line represents data from an individual mouse. OD = optical density. (**C**) Representative images from spleen and liver sections stained by immunohistochemistry to detect STm (blue), Ly6G (brown), or F4/80 (brown). One representative experiment is shown. Arrows indicate positive STm staining. The images on the right are magnifications of the area inside the red-dotted box. Secondary scale bars were added to the original images to ensure consistency throughout the figure. WP = white pulp and RP = red pulp. Data shown as mean ± SD. Two-tailed one-way analysis of variance.

After establishing that macrophages are the primary cell type in the spleen and liver that capture STm, we examined the consequences of macrophage depletion by treating mice with clodronate liposomes. One dose of clodronate is enough to significantly decrease the total number of red pulp macrophages, Ly6C^+^ monocytes, and CD169^+^ metallophilic macrophages in the spleen (15), as well as to deplete the population of Kupffer cells in the liver (Fig. S3). Mice were immunized with OMVs for 12 days to allow normal antibody responses to develop. Then, OMV-immunized mice were treated with control PBS liposomes or clodronate liposomes, and 24 h later, all mice were infected with STm. Untreated controls were mice that were challenged but not vaccinated or liposome treated. Surprisingly, OMV-vaccinated mice treated with clodronate liposomes exhibited similar bacterial burdens to immunized mice treated with PBS liposomes (Fig. 3A). All immunized mice had lower bacterial burdens than untreated mice. Moreover, clodronate liposome treatment did not affect the total anti-OMV IgG levels detected since anti-IgG responses were similar between both groups of OMV-immunized mice (Fig. 3B). The distribution of bacteria in the spleen was then examined using immunohistology. In both non-immunized and OMV-immunized mice treated with control liposomes, bacteria were found associated with macrophages in the red pulp and were rarely observed in the white pulp (Fig. 3C). In clodronate liposome-treated, OMV-vaccinated mice, STm was abundant in the white pulp, typically associated with the follicular dendritic cell network (Fig. 3C). In the liver, bacteria were associated with macrophages in untreated and OMV-immunized mice treated with control liposomes. After clodronate treatment and the consequent absence of monocytic cells in the liver, STm remained detectable in this organ (Fig. 3C). Thus, STm capture by splenic and hepatic macrophages is not necessary for bacterial control but helps restrict dissemination after immunization.

### C1q, C4, and C5, but not C3, are redundant for antibody induction to OMV

**Fig 4:**
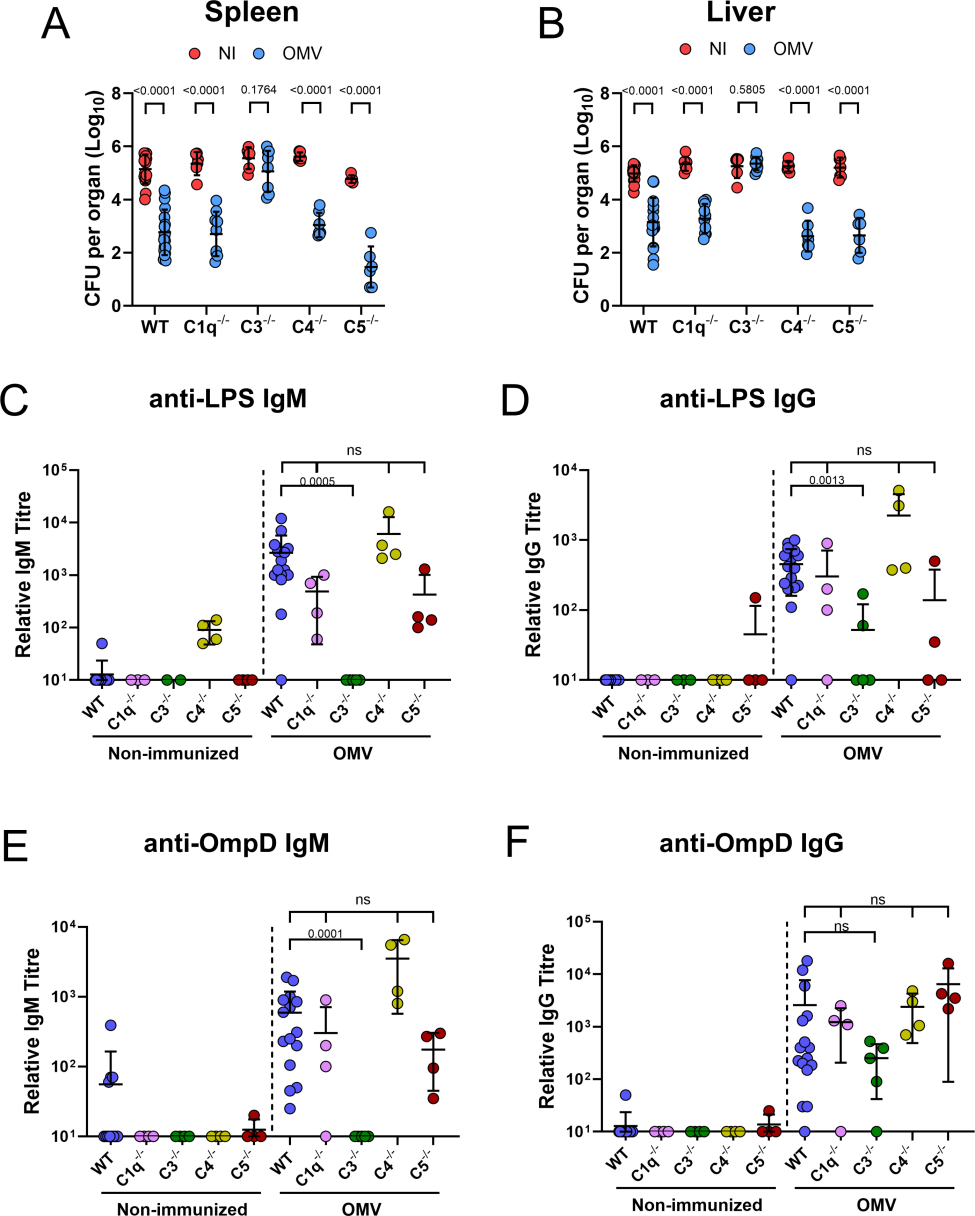
C1q, C4, and C5, but not C3, are redundant for antibody induction to OMV. Non-immunized and OMV-immunized WT, C1q^−/−^, C3^−/−^, C4^−/−^, and C5^−/−^ mice were challenged with STm for 24 h. Bacterial burdens in (**A**) spleen and (**B**) liver. Serum anti-STm LPS IgM (**C**) and IgG (**D**), serum anti-STm OmpD IgM (**E**) and IgG (**F**). Each point shows values from a single mouse. The data shown are from at least two combined experiments. Non-immunized C1q^−/−^ *n* = 6, C3^−/−^ *n* = 6, C4^−/−^ *n* = 6, C5^−/−^ *n* = 5, and WT *n* = 11. OMV-immunized C1q^−/−^ *n* = 9, C3^−/−^ *n* = 9, C4^−/−^ *n* = 7, C5^−/−^ *n* = 6, and WT *n* = 18. Data shown as mean ± SD. A and B were analyzed with a two-tailed unpaired *t*-test. Panels C–F were analyzed with a two-tailed Mann-Whitney *U* test. ns = non-significant.

*In vitro*, mouse complement does not kill *Salmonella* (16). However, C3 has been shown to play a role in antibody-mediated control of infection *in vivo* (8). To examine these potentially conflicting roles of complement in greater depth, mice deficient in specific complement components (C1q, C3, C4, or C5) were immunized and challenged with STm for 24 h. OMV-immunized C1q, C4, and C5-deficient mice exhibited reduced bacterial burdens in the spleen and liver compared to their non-immunized controls (Fig. 4A and B). In contrast, the bacterial burden in the spleens and livers of C3-deficient mice was similar between the immunized and non-immunized groups, indicating that the protection afforded by immunization with OMV was lost in the absence of C3 (Fig. 4A and B). To assess how these different complement-deficient mice responded to OMV and whether the reduced bacterial control in C3 mice was associated with failure to induce antibodies or a loss of antibody function, the levels of anti-lipopolysaccharide (LPS) and anti-OmpD antibodies were measured. In most non-immunized mice, anti-LPS and anti-OmpD antibodies were not detectable, except for C4-deficient mice, which had some IgM anti-LPS antibodies, and three wild-type (WT) mice that had some anti-OmpD IgM antibodies. After immunization with OMV, C1q, C4, and C5 mice all had significant IgM, IgG, and IgG subclass responses to STm antigens, whereas in C3-deficient mice, these responses were absent or minimal (Fig. 4C through F and Fig. S4).

### Anti-STm antibodies reconstitute bacterial control in the absence of C3

**Fig 5:**
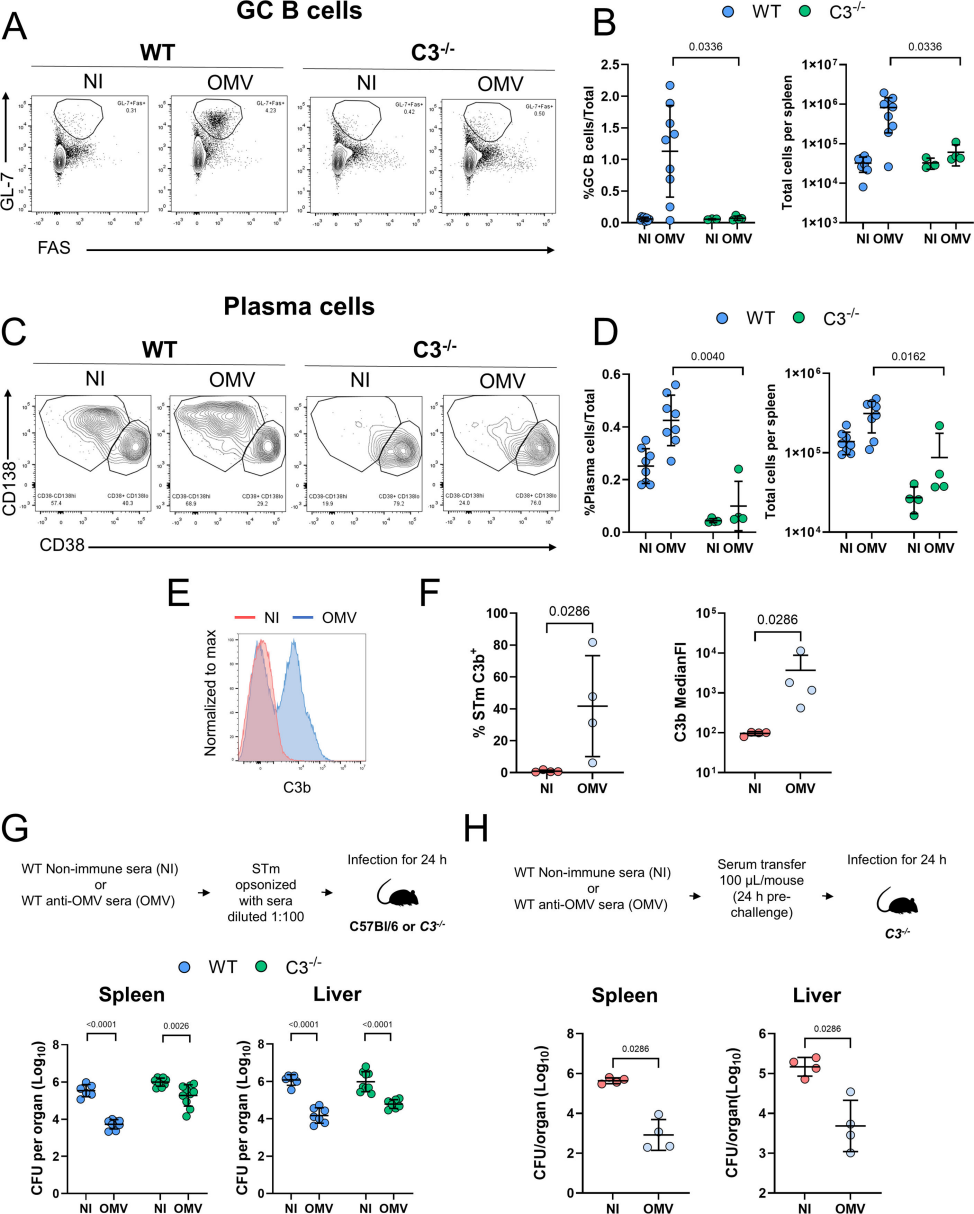
Anti-STm antibodies reconstitute bacterial control in the absence of C3. Splenocytes from non-immunized (NI) and OMV-immunized (OMV) C3^−/−^ and WT mice were examined by flow cytometry. (**A**) Representative flow cytometry plots of germinal center (GC) B cells (GL-7^+^FAS^+^) in NI or OMV-immunized mice. (**B**) Frequency from total cells and total numbers of GC B cells per spleen from panel **A**. (**C**) Representative flow cytometry plots of plasma cells (CD138^+^CD38^−^). (**D**) Frequency from total cells and total numbers per spleen of plasma cells from (**C**). WT NI *n* = 8, C3^−/−^ NI *n* = 4, WT OMV *n* = 9, and C3^−/−^ OMV *n* = 4. (**E**) Representative flow cytometry histogram showing C3b deposition on the surface of STm incubated with sera from NI (red) or OMV (blue) sera. (**F**) Percentage of STm positive for C3b (left) and median fluorescence intensity of C3b (right). (**G**) WT and C3^−/−^ mice were infected for 24 h with STm opsonized with heat-inactivated NI serum or anti-OMV mouse serum (1:100 dilution). The graph shows the bacterial burdens in the spleen and liver. NI→WT *n* = 6, OMV→WT *n* = 8, NI→C3^−/−^ *n* = 9, OMV→C3^−/−^ *n* = 10. (**H**) C3^−/−^ mice received 100 μL of heat-inactivated NI or OMV sera. Twenty-four hours later, all mice were infected with STm for a further 24 h. The graphs show the bacterial burdens in the spleen and liver. NI *n* = 4, OMV *n* = 4. Each point shows data from a single mouse. The data shown are combined from two experiments, except for panels E and F, which are representative of one experiment. Data shown as mean ± SD. (**B, D, and F**) Two-tailed Mann-Whitney *U* test. (**F and H**) Two-tailed unpaired *t*-test.

The impaired antibody responses in C3-deficient mice suggested a defect in B cell responses to OMV immunization. To test this, B cell responses in the spleens of WT and C3-deficient mice were analyzed. After OMV immunization, no significant increase in the frequency or total numbers of germinal center B cells (GC) and plasma cells in C3-deficient mice was observed (Fig. 5A through D). These results suggest that C3 is necessary for the development of protective antibody responses to OMV. Binding of murine antibodies, including anti-OMV antibodies to STm, results in C3b deposition on the surface of the bacteria (Fig. 5E and F; [16]). To determine whether OMV-specific antibodies could reconstitute bacterial control in C3-deficient mice, WT and C3-deficient mice were infected with STm opsonized with either non-immune or anti-OMV immune sera (Fig. 5G). WT mice infected with bacteria opsonized with OMV-specific sera had approximately a median 30-fold lower bacterial burden in the spleen and a 100-fold lower bacterial burden in the liver compared to mice infected with STm opsonized with control sera. In C3-deficient mice, the median differences in the spleen were approximately threefold and fourfold in the liver (Fig. 5G). Opsonization experiments inherently involve lower antibody concentrations, as the sera are diluted 1:100 before use. Thus, we questioned whether C3-deficient mice could control the infection better if antibody levels were less limited. To investigate this, we adoptively transferred non-diluted, heat-inactivated sera (HIS) from OMV-immunized mice into C3-deficient mice and infected them 24 h later (Fig. 5H). In this case, the median fold reduction was 100-fold in the spleen and 25-fold in the liver. Taken together, our results show that C3 can contribute to the antibody-mediated control of STm infection *in vivo* but plays a diminishing role when antibody levels are less limited.

### Anti-STm antibodies and complement cooperate to enhance bacterial uptake in a human cell line *in vitro*

**Fig 6:**
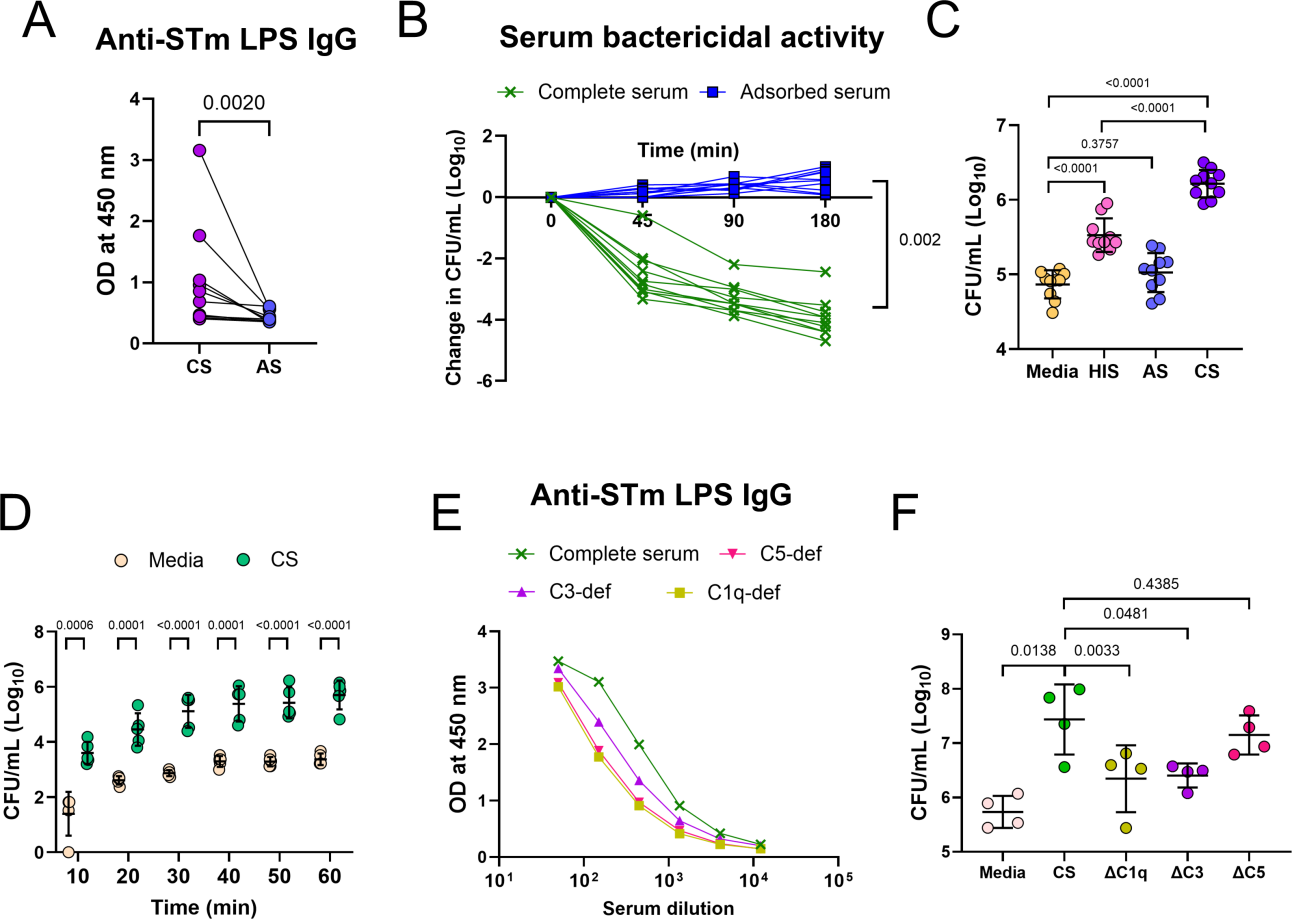
The complement system cooperates with antibodies to promote bacterial uptake by THP-1 cells. (**A**) Anti-STm LPS IgG in sera from healthy individuals in normal serum (CS, *n* = 10) and after adsorption with STm (AS, *n* = 10). Sera were diluted 1:120. (**B**) Serum bactericidal activity of CS (*n* = 10) or AS (*n* = 10) from panel **A**. Each line represents a different donor. (**C**) Intracellular STm in THP-1 cells after incubation with STm and media only, HIS (heat-inactivated sera), AS, or CS, *n* = 10. Further experimental details are presented in Fig. S5A. Each point shows data from a different donor. (**D**) Intracellular STm in THP-1 cells after incubation with STm and media only, or STm with CS. (**E**) Anti-STm LPS IgG in commercial sera deficient of C1q, C3, C5, and CS. (**F**) Intracellular STm in THP-1 cells after incubation with STm and media only, CS, C1q-deficient sera, C3-deficient sera, or C5-deficient sera. Each point shows data from one independent experiment (*n* = 4). Data shown as mean ± SD. Panels A and B were analyzed with a Wilcoxon matched-pairs signed rank test. Panels C and F were analyzed with a two-tailed one-way analysis of variance test. Panel D was analyzed with an unpaired *t*-test.

To examine whether the features associated with murine macrophages *in vivo* can also be observed in human-derived cells *in vitro*, we infected differentiated THP-1 cells with STm in the presence or absence of human serum. This takes advantage of the observation that nearly all human adults possess bactericidal antibodies against *Salmonella* (17, 18). All sera used in these experiments had bactericidal activity that was lost after absorbing STm-specific antibodies after mixing with whole STm (Fig. 6A and B). Additionally, all sera used had normal complement activity, as assessed by the CH50 assay (Fig. S5B). Infection of THP-1 cells with bacteria opsonized with complete sera (CS) containing both complement and antibodies led to the highest uptake of STm (Fig. 6C). When sera that were heat inactivated to destroy complement but had antibodies were used, bacterial uptake was increased, but this was not as high as for CS. In contrast, no increase in bacterial numbers was detected when adsorbed sera (AS) were used, indicating that complement alone is not sufficient to promote bacterial uptake in this system (Fig. 6C). In this system, the presence of complete serum enhanced bacterial uptake by 10 min post-infection (Fig. 6D). To test which specific complement component was required, we performed the same experiment using human serum deficient in C1, C3, or C5. Anti-STm IgG antibody levels in these sera were comparable to those observed in sera obtained from healthy volunteers (Fig. 6E). CS and C5-deficient sera promoted bacterial uptake to similar levels. In contrast, bacterial uptake with C1q- or C3-deficient sera was lower than that with CS (Fig. 6F). This finding is consistent with mouse studies showing that antibodies promote bacterial uptake, whereas complement enhances its efficiency. Therefore, *in vitro*, antibodies enhance bacterial uptake by THP-1 cells, while complement also plays a role in promoting this activity.

## DISCUSSION

Antibodies play a crucial role as effector molecules for OMV vaccines (14, 19, 20). Dissecting the *in vivo* mechanisms by which antibodies function is complicated by the intricate dynamics between pathogens and the various components of the immune system. Our study aimed to explore the interactions among the pathogen, antibodies, phagocytes, and the complement system, both *in vivo* and *in vitro*, to identify the key mechanisms underlying pathogen control. Our findings demonstrate that monocytic cells can phagocytose *Salmonella* and that antibodies and complement work together to enhance this process. However, the most critical point from these studies is that, in our models and under the conditions tested, antibodies can be sufficient for protection, while complement and other factors support downstream functions resulting from antibody binding to pathogens. The intrinsic redundancy between these mechanisms underscores the importance of antibody binding for protection and provides avenues to counteract the effectiveness of pathogen escape strategies. This ultimately benefits the host by increasing the likelihood that antibodies will reduce the pathogen burden and prevent disease.

Antibody activity against bacterial infections is often assessed by serum bactericidal activity or opsonophagocytic activity. In recent years, these approaches have been complemented by the development and use of systems serology approaches, which have provided additional, more nuanced insights into the effector functions of antibodies against bacterial pathogens. This enhanced understanding of how antibodies can act against bacteria may help capture the breadth of their activity against specific pathogens. This diversity in antibody function is reflected in the current study in the assessment of responses across multiple organs, both in the presence and absence of selective cell types and complement components. *In vivo*, a striking finding was that in the presence of antibodies, macrophages remained the most efficient cell type associated with STm. This association with macrophages was observed in both the spleen and the liver. Moreover, our infection studies using antibodies and THP-1 cells also demonstrated that antibodies enhanced bacterial uptake. This suggests a functional overlap in these activities between mice and humans in macrophage-lineage cells.

After challenging vaccinated and clodronate-liposome-treated mice, we observed a more widespread bacterial antigen staining pattern. Antigen was readily detected in the splenic white pulp and was particularly associated with the follicular dendritic cell network. This differs markedly from previous reports, in which the antigen is mainly detected in the red pulp areas and not in the follicles (15, 21). Despite this, clodronate-treated mice had similar overall bacterial burdens to those of control mice. This suggests that a significant contribution of macrophages to controlling infection is to contain and limit bacterial spread. This is a vital role of antibodies since controlling bacterial dissemination is a key factor in limiting the risk of severe complications from infections, such as sepsis. For instance, after primary *Salmonella* infection, cardiovascular complications, such as splenic and hepatic thrombosis, occur in infected mice; however, we have not observed this complication in vaccinated and challenged mice. Moreover, by restricting bacterial dissemination, macrophages could help reduce the opportunities for bacteria to reach niches where they can persist and drive inflammation.

Antibody-dependent neutrophil phagocytosis (ADNP) activity is associated with anti-*S*. Typhi Vi responses after vaccination (22), which led to our original hypothesis that in tissues, neutrophils are the primary cells that phagocytose STm after immunization. However, this was not the case. Intravital studies indicated that the predominant localization of bacteria to macrophages was not due to neutrophil uptake immediately after infection. Therefore, neutrophil cell death concurrent with phagocytosis of STm does not appear to contribute to the predominance of STm in macrophages following challenge of vaccinated mice. The reasons why macrophages are the predominant host cell type associated with bacterial uptake post-vaccination in this model remain unclear. Nonetheless, this predominance does not stem from the inability of neutrophils to be infected by *Salmonella*. Previous studies, including those from our own group, have demonstrated that purified neutrophils can be efficiently infected *in vitro* (23). Moreover, we and others have shown that neutrophil depletion has minimal impact on bacterial control after systemic infection with STm (15, 24). Therefore, the role of neutrophils post-vaccination in this model remains unclear. We hypothesize that macrophages outcompete neutrophils in capturing STm, leaving a limited number of bacteria in extracellular spaces available for neutrophils to phagocytose. Meanwhile, vaccine-induced antibodies enhance macrophage phagocytosis, further reducing the opportunities for neutrophils to phagocytize STm. This hypothesis will be tested in future studies.

The C1q, C4, and C5 components of the complement system were not essential for antibody-mediated protection. Non-immunized C1q-deficient mice exhibit a transient increase in bacterial numbers during the initial days of primary STm infections. Still, no further role for C1q in the antibody-mediated control of *Salmonella* infection has been previously assessed (25). Since C1q and C4 are essential components in the classical pathway for generating the C3 convertase, this suggests that the classical pathway is not crucial for reducing bacterial numbers in this model. Additionally, complement effector functions mediated by C5 are not essential for controlling *Salmonella* infection in vaccinated mice, as C5-deficient mice produced normal antibody levels and controlled the infection. Since immune mouse serum does not induce cell-independent bactericidal activity against *Salmonella*, it suggests that antibodies must be functioning through alternative mechanisms to exert bacterial control (16, 26). We did not assess the effects of complement deficiency on the kinetics of antibody-mediated effector mechanisms. In the absence of complement factors, for example, there may be changes in the rate of bacterial uptake into intracellular niches, or the extracellular niches where STm may reside could also differ.

Immunized C3-deficient mice were not protected against infection, likely due to the lower antibody responses induced to OMV in these mice. Although C3-deficient mice had poor antibody responses overall, some IgG to OmpD was observed. Although it is unclear why this response was partially intact, it may be because OmpD, like other trimeric porins, can trigger T-independent extrafollicular responses with switching to IgG (13, 27). Nevertheless, after the adoptive transfer of immune serum into C3-deficient mice and subsequent challenge, protection was observed. Therefore, while C3 is necessary for B cell responses to develop against protective antigens within OMVs, it is not essential for the *in vivo* functionality of anti-STm antibodies in mice. Previously, antibody-opsonized *E. coli* capture by Kupffer cells in the absence of C3 has been reported, but the mechanism by which this occurs remains elusive (28). Work by Rossi and colleagues demonstrated a role for C3 in antibody-mediated protection after the adoptive transfer of a monoclonal IgG2a antibody to the STm LPS O4 antigen. In contrast, antibody responses to OMV consist of multiple isotypes targeting multiple antigens, which may compensate for the need for C3 in providing protection. Indeed, multiple isotypes can contribute to antibody-mediated control of STm infections in mice, as demonstrated by studies using adoptive transfer of monoclonals and IgG1-deficient mice (7, 29, 30). Thus, when multiple antigens are targeted, there may be greater redundancy for complement. Alternatively, the significance of C3 may increase based on antibody availability. For instance, when antibodies are abundant, the role of C3 may be less critical. Conversely, if antibody levels are lower, C3 may be more crucial in facilitating bacterial opsonophagocytosis. This situation may be particularly relevant several months or years after vaccination, as antibody levels naturally decline. The study by Rossi and colleagues (8) showed that Fc gamma receptors (FcγRs) do not play a role in reducing bacterial burden, underscoring the need for further research to fully understand their role in protecting against *Salmonella*. There remains much to understand about how antibodies function *in vivo* against this pathogen.

There are limitations in this study. A model of resolving infection using attenuated STm in susceptible mice was used in most experiments. Further studies involving complement-deficient mice infected with wild-type virulent STm, infected for longer periods, could help to fully understand how antibodies confer protection under different conditions. Additionally, a critical aspect not addressed in our investigation is how antibodies protect at mucosal surfaces, the primary route of infection for many pathogens. Despite this, we can conclude that, under the conditions tested, antibodies against STm can confer protection against infection via multiple mechanisms. Antibody responses to antigenically complex pathogens likely operate through numerous, redundant, and overlapping mechanisms simultaneously. These include antibody-dependent cell cytotoxicity and ADNP, which were not evaluated in this study but are likely to contribute to protection. This suggests that, mechanistically, protection is associated with a signature rather than a single pathway, which has implications for developing correlates of protection for vaccines against bacterial infections.

## MATERIALS AND METHODS

### Mice, bacterial strains, immunogens, and immunization

OMV were prepared from a TolR-deficient *Salmonella* Typhimurium strain 14,028 as described previously (14). Protein content was quantified with a bicinchoninic acid assay kit following the manufacturer’s instructions (Thermo Scientific, Cat. No. 23227). *Salmonella* Typhimurium LPS was purchased from Enzo (Cat. No. ALX-581-011-L002). OmpD from *Salmonella* Typhimurium was purified as described previously (6).

WT C57BL/6J mice were obtained from Charles River UK (strain #27). C1q-deficient mice (31), C3-deficient mice (32), and C5-deficient mice (33) were kindly donated by Marina Botto. Leo C. James kindly donated C4-deficient mice (32). For all experiments, a mix of male and female mice, aged 6–12 weeks, was used. Mice were housed in specific-pathogen-free conditions under a 12 h light/dark cycle and a controlled temperature range of 20°C–24°C. All mice were euthanized by cardiac bleed under anesthesia, followed by cervical dislocation.

Mice were immunized intraperitoneally (i.p.) with 1 µg of OMV. PBS-injected mice were used as non-immunized controls. Fourteen days post-immunization, mice were injected i.p. with 5 × 10^5^ CFU of a *Salmonella* Typhimurium Δ*aroA* SL3261 strain (33). Clodronate-depletion experiments were performed as described previously (34, 35). Briefly, WT mice were immunized with OMVs as described above. Twelve days post-immunization, mice were injected i.v. with 200 µL of clodronate liposomes (Clodrosome, CLD-8909) or PBS liposomes (Encapsome, CLD-8901, both from Encapsula NanoSciences). After 24 h, mice were infected with STm as described above. Unvaccinated mice infected with STm that were not injected with liposomes served as untreated controls. For opsonization experiments, 5 × 10^5^ bacteria/mL were mixed for 30 min with pooled heat-inactivated anti-OMV sera (diluted 1:100) or pooled heat-inactivated non-immune sera (diluted 1:100). Mice were then infected with 5 × 10^5^ opsonized bacteria via i.p. Opsonized bacteria were plated onto LB agar plates to confirm that the opsonization did not affect viability. For passive transfer experiments, C3-deficient mice were injected intravenously with 100 µL of pooled heat-inactivated non-immune or anti-OMV sera prepared from mice immunized twice, 35 days apart, with 1 µg of OMV. Twenty-four hours later, the mice were infected as described above.

### Intravital microscopy

Male C57Bl/6 mice were vaccinated with OMVs as previously described. Fourteen days post-immunization, the mice were anesthetized using a combination of ketamine hydrochloride (Anesketin, Dechra Veterinary Products Inc.) at a dosage of 200 mg/kg and xylazine (Nerfasin 100, Dechra Veterinary Products Inc.) at 10 mg/kg. The liver and spleen were then exposed and imaged following established protocols (15). Anti-mouse F4/80 (1.6 µg, clone BM8, BioLegend) and anti-Ly6G (1.6 µg, clone 1A8, BioLegend) were injected i.v. 30 min before imaging. Once the recording was started, mice were challenged intravenously with approximately 10^7^ CFU of *Salmonella* Typhimurium expressing EGFP (36). Ten-minute-long videos were recorded using an inverted Leica SP8 microscope (Leica Microsystems). Image analysis was performed with Fiji (version 2.9.0).

### Removal of anti-*Salmonella* antibodies from serum and serum bactericidal assay

Serum was collected from healthy individuals by venipuncture and stored at −80°C until needed (Ethical approval: ERN_1805 December 2023). C1q (Cat. No. A509), C3 (Cat. No. A508), and C5-depleted sera (Cat. No. A501) were obtained from QuidelOrtho. To remove specific STm antibodies, 900 µL of human serum was incubated with 100 µL of a hyper-concentrated suspension of washed STm D23580 at 4°C for 1 h. After centrifugation at 6,000 × *g* for 5 min, the supernatant was further adsorbed two times. The adsorbed serum was aliquoted and stored at −80°C until needed. For the serum bactericidal assay, approximately 10^5^ CFU of mid-log STm D23580 were incubated with complete serum or adsorbed serum. The samples were incubated in a shaking incubator at 37°C, and aliquots were taken at 45, 90, and 180 min. Each aliquot was diluted 10-fold and plated on LB agar to quantify CFU per milliliter. The change in the CFU was determined by subtracting the base 10 logarithm of the CFU at the specified time point from the base 10 logarithm of the CFU at time zero.

### Statistical analysis

All experiments were conducted at least twice. In all cases, group sizes were determined using G*Power 3.1.9.2 based on expected group differences to achieve at least 80% power and a *P* < 0.05 significance level. Age-matched mice of the same genotype were randomized between groups. Colony-forming unit counts were expressed as the base 10 logarithm to approximate a normal distribution and were analyzed using a two-tailed unpaired *t*-test for comparisons between two groups, or a two-tailed one-way analysis of variance for comparisons involving more than two groups (Fig. 3A, 4A and B, 5F and H, 6C, D and F). For the remaining analyses, no normality assumptions were made. Thus, the data were analyzed using nonparametric tests (two-tailed Mann-Whitney *U* test or Wilcoxon matched-pairs signed rank test for Fig. 6A and B). All *P*-values were calculated with GraphPad Prism version 10.1.0 and were considered significant when *P* ≤ 0.05.

## Data Availability

Additional data are available from A.F.C. upon reasonable request.

## SUPPLEMENTAL MATERIAL

Legends mbio.02846-25-s0001.docx Video legends.

Supplemental material mbio.02846-25-s0002.pdf Supplemental figures and text.

Table S1 mbio.02846-25-s0003.xlsx List of antibodies and reagents.

Table S2 mbio.02846-25-s0004.xlsx OptiVaNTS member details.

Video S1 mbio.02846-25-s0005.mp4 Non-immunized spleen.

Video S2 mbio.02846-25-s0006.mp4 OMV-immunized spleen.

Video S3 mbio.02846-25-s0007.mp4 Non-immunized liver.

Video S4 mbio.02846-25-s0008.mp4 OMV-immunized liver.

Video S5 mbio.02846-25-s0009.mp4 Non-immunized liver 6 h post-infection.

Video S6 mbio.02846-25-s0010.mp4 OMV-immunized liver 6 h post-infection.

ASM does not own the copyrights to Supplemental Material that may be linked to, or accessed through, an article. The authors have granted ASM a non-exclusive, world-wide license to publish the Supplemental Material files. Please contact the corresponding author directly for reuse.
