# Supplementary material for: Vaccine-induced antibodies can limit Salmonella infection in the absence of complement or macrophages
Source: mBio. 2026 Feb 25;17(4):e02846-25. doi: 10.1128/mbio.02846-25 (PMC13059751; doi:10.1128/mbio.02846-25)
Supplement: Legends — Video legends. [file mbio.02846-25-s0001.docx]

**Video S1.** Representative video of the spleen from a non-infected mouse. Mice were prepared for intravital microscopy as outlined in the methods section. Approximately 10^7^ CFUs of GFP-STm were injected intravenously at time zero. The time-lapse video shows the elapsed time in minutes, seconds, and milliseconds. Red= Ly6G, blue= F4/80, green= STm.

**Video S2.** Representative video of the spleen from a mouse immunized with STm OMVs. Mice were vaccinated with 1 µg of OMVs for 14 days and prepared for intravital microscopy as outlined in the methods section. Approximately 10^7^ CFUs of GFP-STm were injected intravenously at time zero. The time-lapse video shows the elapsed time in minutes, seconds, and milliseconds. Red= Ly6G, blue= F4/80, green= STm.

**Video S3.** Representative video of the liver from a non-immunized mouse. Mice were prepared for intravital microscopy as outlined in the methods section. Approximately 10^7^ CFUs of GFP-STm were injected intravenously at time zero. The time-lapse video shows the elapsed time in minutes, seconds, and milliseconds. Red= Ly6G, blue= F4/80, green= STm.

**Video S4.** Representative video of the liver from a mouse immunized with STm OMVs. Mice were vaccinated with 1 µg of OMVs for 14 days and prepared for intravital microscopy as outlined in the methods section. Approximately 10^7^ CFUs of GFP-STm were injected intravenously at time zero. The time-lapse video shows the elapsed time in minutes, seconds, and milliseconds. Red= Ly6G, blue= F4/80, green= STm.

**Video S5.** Representative video of the liver from a non-immunized mouse infected i.p. for 6 hours with approximately 10^6^ CFU GFP-STm. The time-lapse video shows the elapsed time in minutes, seconds, and milliseconds. Red= Ly6G, blue= F4/80, green= STm.

**Video S6.** Representative video of the liver from a mouse immunized with OMVs. Mice were immunized with OMVs and 14 days later challenged with approximately 10^6^ CFU GFP-STm i.p. for 6 hours. The time-lapse video shows the elapsed time in minutes, seconds, and milliseconds. Red= Ly6G, blue= F4/80, green= STm.
