# Supplementary material for: Vaccine-induced antibodies can limit Salmonella infection in the absence of complement or macrophages
Source: mBio. 2026 Feb 25;17(4):e02846-25. doi: 10.1128/mbio.02846-25 (PMC13059751; doi:10.1128/mbio.02846-25)
Supplement: Supplemental material — Supplemental figures and text. [file mbio.02846-25-s0002.pdf]

Vaccine-induced antibodies can limit Salmonella infection in the absence of complement or macrophages.

Marisol Perez-Toledo1, Kubra Aksu-Istil, Edith Marcial-Juarez, Ruby R Persaud, Areej Alshayea, Sian E. Jossi, Agostina Carestia, Fien von Meijenfeld, Marina Botto, Leo C James, Bas Surewaard, Zaheer Afzal, Adrian M Shields, William Horsnell, Constantino Lopez-Macias, Ian R Henderson, OptiVaNTS consortium, Craig N Jenne, Adam F Cunningham

***Correspondence:** Marisol Perez-Toledo and Adam F Cunningham
email: m.perez-toledo@bham.ac.uk, a.f.cunningham@bham.ac.uk;

Supplementary figures and information

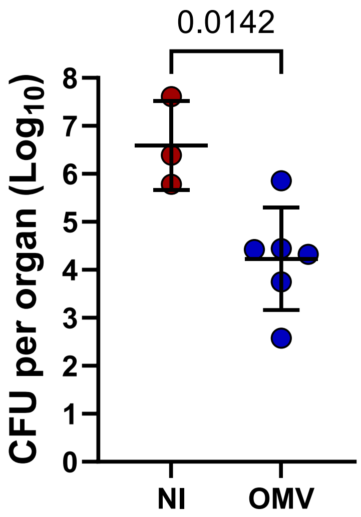

Figure S1. Immunization with OMVs reduces the bacterial burden in the spleen upon challenge with STm LT2. WT mice were immunized with 1 µg of STm OMVs. On day 14, mice were infected i.p. with 10^7 CFU of STm LT2 for 6 hours. Non-immunized (NI) mice were used as controls. Data shown as mean ± SD. Each point represents an individual mouse. Two-tailed unpaired t-test. *P<0.05.

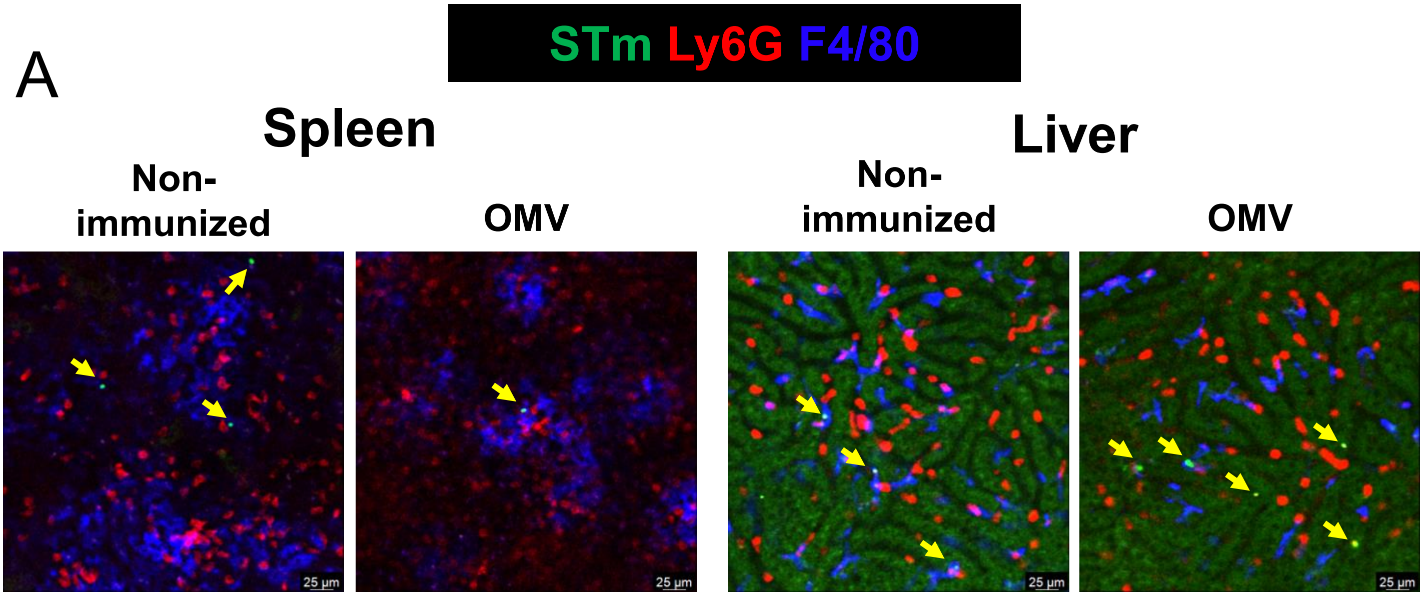

Figure S2. STm is found associated with F4/80⁺ cells in the spleen and liver 6 hours post-infection, regardless of vaccination status. WT mice were immunized with 1 µg of STm OMVs. On day 14, mice were infected i.p. with 10^7 CFU of STm LT2 for 6 hours. Mice were prepared for live imaging as described in the methods. 30 minutes before imaging, mice were injected i.v. with anti-mouse Ly6G (red) or anti-F4/80 (blue). Yellow arrows indicate the presence of STm.

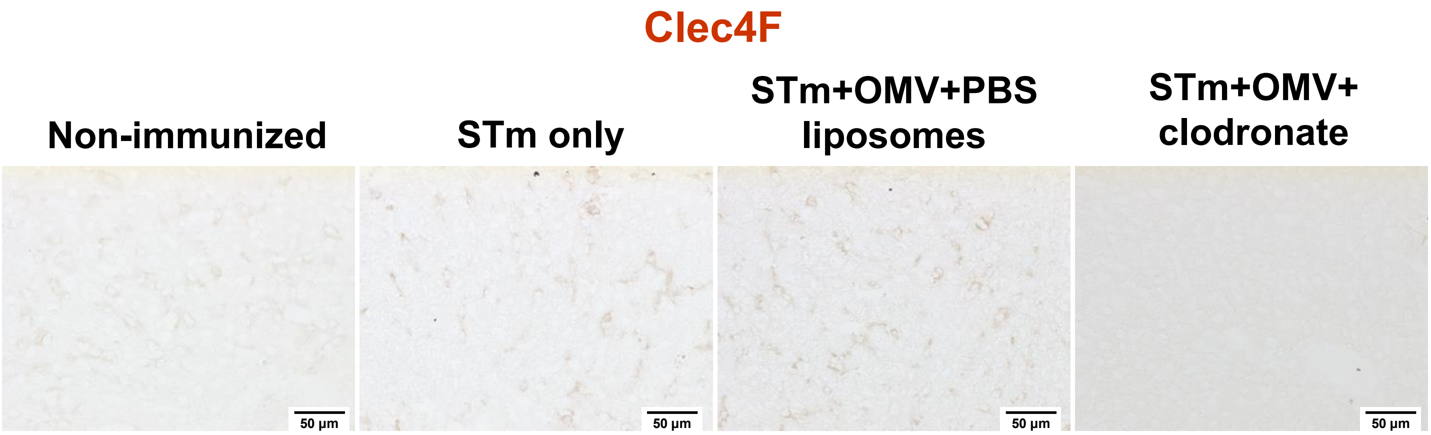

Figure S3. Representative immunohistochemistry images of sections stained for Clec4F (Brown). Liver microsections from C57BL/6 mice that were non-immunized, or received STm only, or were immunized with OMVs and treated with control PBS liposomes and challenged with STm, or immunized with OMVs, treated with clodronate liposomes and infected with STm.

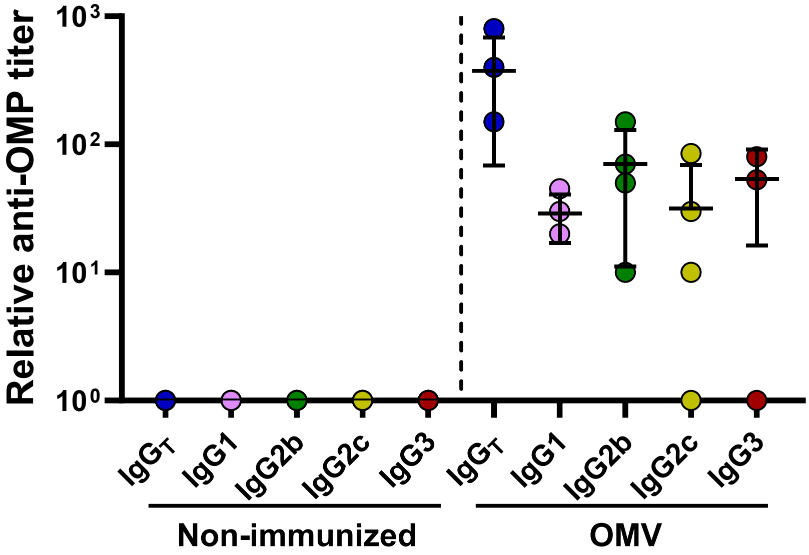

Figure S4. Immunization with OMVs induces all IgG isotypes. Serum total IgG (IgG_T) and IgG isotypes (IgG1, IgG2b, IgG2c, and IgG3) against Outer Membrane Proteins (OMP) were determined by ELISA. WT mice were immunized with STm OMVs for 28 days. Non-immunized mice were included as controls. Each point depicts one individual mouse. Mean ± SD is shown. Representative experiment with n=4 mice per group.

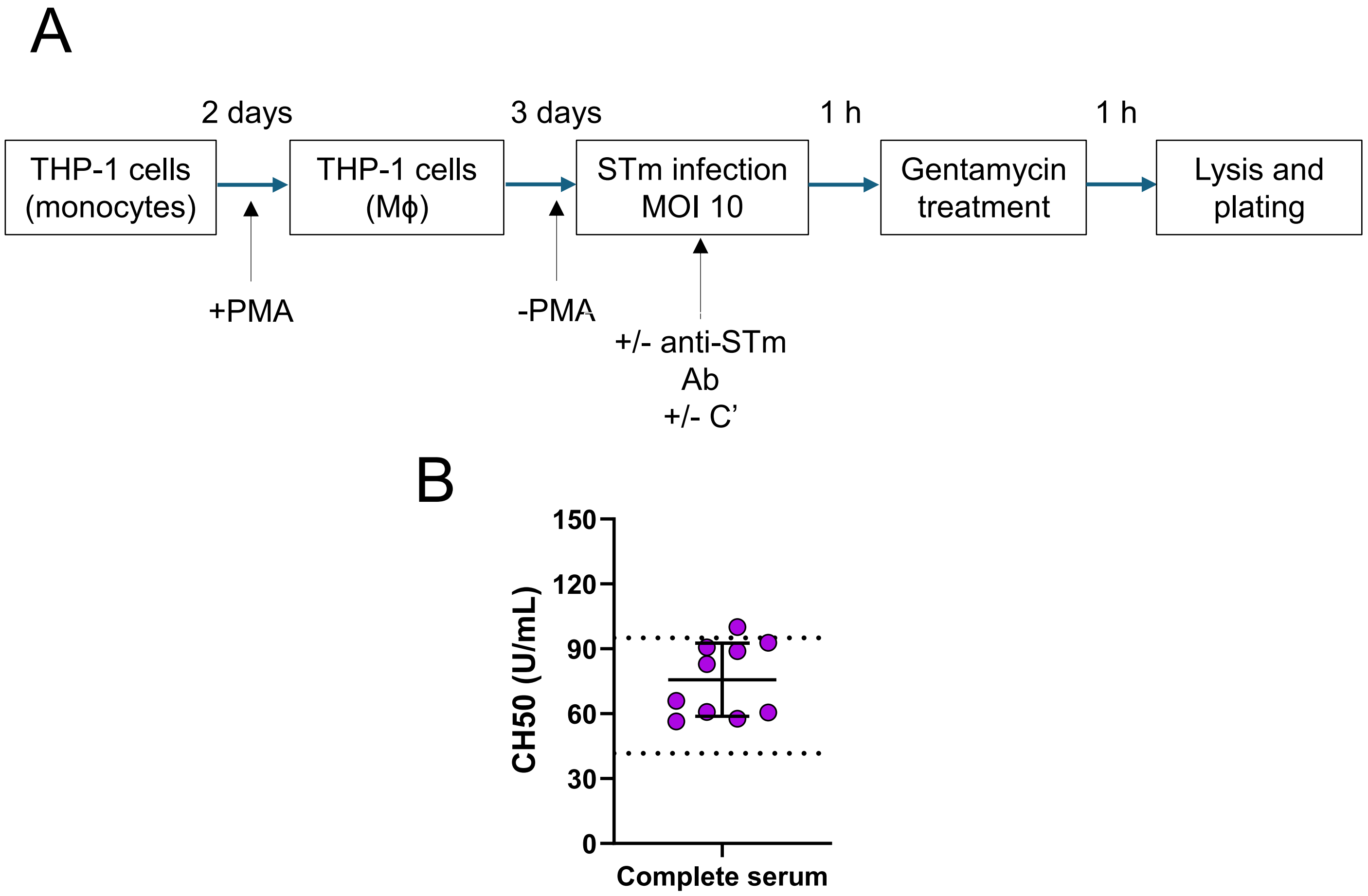

Figure S5. (**A**) THP-1 cells were differentiated with PMA for 2 days and then rested without PMA for an additional 3 days. Following this, the cells were infected with *Salmonella* Typhimurium (STm) at a multiplicity of infection (MOI) of 10, either in the presence or absence of anti-STm antibodies and complement. After a 1-hour incubation period, the cells were treated with gentamicin for another hour. Subsequently, the cells were lysed, diluted, and plated for colony-forming unit (CFU) counting. (**B**) Complete Hemolytic Activity (CH50) in the serum samples used in Figure 6A-C. The horizontal dotted lines show the 95th Percentile range reported by the manufacturer. Mean±SD is shown. Each point represents one donor (n=10).

Preparation of STm for infection and colony-forming units quantification

To prepare the bacteria for challenge, one colony of STm SL3261 grown on Luria-Bertani (LB) agar was inoculated into 10 mL of LB broth and incubated overnight at 37 °C. The following day, the bacteria were regrown in fresh LB medium with shaking at 180-200 rpm and 37 °C until the optical density at 600 nm reached approximately 1.0. 1 mL of the culture was centrifuged at 6000 g and washed twice with sterile PBS. The final pellet was resuspended in 1 mL of PBS and diluted to the final injection concentration. Serial dilutions of the leftover bacterial preparation confirmed the administered bacterial dose. Mice were sacrificed 24 hours post-challenge. Livers and spleens were collected to quantify the bacterial burden in organs. Approximately 1 mg of tissue was homogenized through a 70-μm cell strainer and reconstituted in 1 mL of sterile PBS. Neat and serial 1:10 dilutions were plated on LB agar plates and incubated at 37 °C overnight for CFU counting.

Immunohistology

5 μm cryosections of spleens and livers were fixed in acetone (Acros Organics Cat. No. 268310025) for 20 min and then stored at -20 ºC until analysis. For immunohistochemistry, the sections were rehydrated in Tris-buffered Saline (pH 7.6) at room temperature and stained in the same buffer with primary antibodies for 45 minutes. HRP-conjugated or biotin-conjugated secondary antibodies, along with the Vectastain® ABC-AP alkaline phosphatase kit (Vector Laboratories Cat. No. Ak-5000), were used as secondary reagents. HRP activity was detected with SIGMA*FAST* 3-3'Diaminobenzidine tablets (Sigma-Aldrich Cat. No. D4293), while alkaline-phosphatase activity was detected using naphtol AS-MX phosphate and fast blue salt, with levamisole (All from Sigma-Aldrich).

For immunofluorescence, the sections were re-hydrated in PBS (pH 7.4) and blocked for 10 minutes with 10% fetal bovine serum (FBS) in PBS. Additional biotin-blocking steps were performed for the liver prior to staining using an avidin/biotin blocking kit (Vector Laboratories Cat. No. SP-2001) following the manufacturer's instructions. Antibodies were

incubated in the dark at room temperature for 40 minutes. Slides were then mounted in Prolong Diamond (ThermoFisher Cat. No. P36970) and allowed to cure for 24 hours at room temperature in the dark before imaging. Images were acquired with a Zeiss Axio Scan Z1 slide scanner (Zeiss, Germany). A detailed list of primary and secondary antibodies used is provided in Table S1.

ELISA

96-well Nunc Maxisorb plates (ThermoFisher Scientific, Cat No.442404) were coated with either STm LPS, STm OmpD, or STm OMVs at 5 µg/mL diluted in carbonate buffer. After blocking for 1 hour at room temperature with 2% bovine serum albumin (BSA) (Sigma, Cat. No. A7906-100G), mouse serum was added at a 1:50 or 1:100 dilution, then diluted three-fold. After incubation for one hour at 37 °C and washing with PBS-0.001% Tween 20, AP-conjugated anti-mouse IgM (1:2000), IgG (1:1000), IgG1 (1:1000), IgG2b (1:1000), IgG2c (1:1000), and IgG3 (1:1000, all from SouthernBiotech, details in Table S1) were added to the wells, followed by a further one-hour incubation at 37 °C. After washing with PBS-0.001% Tween 20, development was performed with Sigma FAST™ p-Nitrophenyl Phosphate tablets (Cat. No. N2770), prepared according to the manufacturer's instructions, and the absorbance was measured at 405 nm approximately 1 hour later. Titers were calculated by identifying the dilution at which the OD_{405} was equal to 2.

Sample preparation for flow cytometry

Sample preparation for flow cyometry analysis was performed as described before (1). Briefly, single-cell suspensions from spleens were obtained by mashing approximately 20 mg of tissue through 50 µm cell strainers (CellTrics Cat. No. 04-0042-2317) in 5 mL of RPMI 1640 (Gibco Cat. No. 31870-025) supplemented with 5% FBS (Gibco Cat. No. A5256801) and 5 mM EDTA (PanReac AppliChem Cat. No. A4892). Red blood cells were lysed with ACK lysis buffer (Life Technologies, Cat. No. A10492-01), and after washing, the cell suspension was adjusted to 10^7 cells/mL. 2.5×10^6 cells were then incubated with an anti-

CD16/32 antibody (Invitrogen Cat. No. 14-01-61-85) in FACS buffer (2% FBS, 5 mM EDTA and 0.01% sodium azide). Live/Dead staining was performed with ZombieAqua™ or ZombieViolet™, according to the manufacturer's instructions (BioLegend Cat. Nos. 423101 and 423113). Cell suspensions were incubated on ice for 25 minutes with a mix of the primary antibodies in FACS buffer. Samples were acquired using a BD LSR II Fortessa flow cytometer, and data were analyzed using Flowjo Software v10.8.1 (BD Life Sciences). Detailed information on the antibodies used is provided in Table S1.

Image analysis

Image analysis was conducted in Fiji. To determine the frequency of STm+ pixels in Ly6G or F4/80 cells, four different fields of view were captured from each spleen and liver. For each image, a Gaussian blur (sigma=1) and background subtraction were applied. Then, each image was split into green (STm), blue (F4/80), and white (Ly6G) channels. Pixels present in both STm and F4/80, or STm and Ly6G, were identified using the Boolean operator “AND” in the Image Calculator function. The resulting image was binarized with auto threshold, and the Analyze Particles function was used to quantify the pixel area.

To quantify the total number of STm per field of view, GFP-only snapshots from specific time points were taken, and the number of particles was measured using the Analyze Particles function. To determine the total number of STm captured per field of view, GFP-positive events that remained stationary (moving less than 1 cell diameter for ≥3 minutes) were manually counted. The number was expressed as a percentage, with the total number of STm at a specific time set to 100%. To assess the association of STm with either F4/80 or Ly6G cells, a snapshot of the last frame of each video was analyzed. The image was split into green (STm), blue (F4/80), and white (Ly6G) channels. Then, an image showing the pixels present in both STm and F4/80, or STm and Ly6G, was produced using the Boolean AND operator in the Image Calculator function. This resulting image was converted to binary using auto threshold, and the Analyze Particles function was used to quantify the pixel area. The results are expressed as a percentage, where the total number of STm in the same

frame is set to 100%. To analyze the particle movement, time-lapse stacks were analyzed with TrackMate (2).

In vitro gentamicin protection assay with THP-1 cells

THP-1 cells were obtained from ATCC (TIB-202). Cells were cultured in RPMI-1640 complete medium containing 10% FBS (Gibco Cat. No. A5256801), 1% L-glutamine (Gibco Cat. No. A5256801), and 1% penicillin-streptomycin (Gibco 15140-122) at 37 °C with 5% CO_2. Differentiation of THP-1 cells was performed by incubating them with 200 ng/mL Phorbol 12-Myristate 13-Acetate (Sigma-Aldrich, Cat. No. P8139) for 2 days, followed by a rest period of 3 days. 2 days before the experiment, cells were washed once in PBS, resuspended at a density of $2x10^5$ cells/mL, and seeded in 96-well plates. Cells were infected with STm strain D23580 (3), at a multiplicity of infection (MOI) of 10. Before infection, the bacteria were opsonized for 10 minutes with human serum at a 1:10 dilution. The infection was performed for 1 hour, followed by a further 1-hour incubation in the presence of gentamicin (Gibco Cat. No. 15710-064) at 100 µg/mL. The bacterial count was determined by lysing the cells with 0.2% deoxycholate, followed by serial 10-fold dilutions in PBS. Dilutions were plated in LB agar plates and incubated overnight at 37 °C.

C3b deposition assay

Salmonella Typhimurium ΔaroA SL3261-GFP (4) was cultured overnight in Luria Bertani broth containing 100 µg/mL ampicillin. The bacteria were washed twice with PBS, and 50 µL of the bacterial suspension was incubated with 50 µL of serum diluted 1:50 in PBS. After 30 minutes of incubation at 37 °C, the suspension was washed with PBS. Then, 50 µL of 10% normal mouse serum (diluted in PBS with 5 mM $MgCl_2$ and 5 mM $CaCl_2$) was added, and the mixture was incubated for 30 minutes at 4 °C. After washing, a PE-conjugated anti-C3b (clone 6C9/C3b, Biolegend, Cat. No. 846502) was added at 10 µg/mL. The mixture was incubated for 30 minutes on ice, washed, and resuspended in PBS with 1%

paraformaldehyde for acquisition on a Beckman-Coulter CytoFLEX flow cytometer and analysis with FlowJo v10.8.1 (BD Life Sciences).

Determination of total classical complement activity

The total classical complement activity (CH50) of the serum samples was determined using the Optilite® CH50 Reagent (The Binding Site, product code NK095.OPT) according to the manufacturer's instructions.

References

1. Marcial-Juarez E, Perez-Toledo M, Nayar S, Pipi E, Alshayea A, Persaud R, Jossi SE, Lamerton R, Barone F, Henderson IR, Cunningham AF. 2023. Salmonella infection induces the reorganization of follicular dendritic cell networks concomitant with the failure to generate germinal centers. iScience 26:106310.

2. Tinevez JY, Perry N, Schindelin J, Hoopes GM, Reynolds GD, Laplantine E, Bednarek SY, Shorte SL, Eliceiri KW. 2017. TrackMate: An open and extensible platform for single-particle tracking. Methods 115:80-90.

3. Kingsley RA, Msefula CL, Thomson NR, Kariuki S, Holt KE, Gordon MA, Harris D, Clarke L, Whitehead S, Sangal V, Marsh K, Achtman M, Molyneux ME, Cormican M, Parkhill J, MacLennan CA, Heyderman RS, Dougan G. 2009. Epidemic multiple drug resistant Salmonella Typhimurium causing invasive disease in sub-Saharan Africa have a distinct genotype. Genome Res 19:2279-87.

4. Flores-Langarica A, Marshall JL, Bobat S, Mohr E, Hitchcock J, Ross EA, Coughlan RE, Khan M, Van Rooijen N, Henderson IR, Maclennan IC, Cunningham AF. 2011. T-zone localized monocyte-derived dendritic cells promote Th1 priming to Salmonella. Eur J Immunol 41:2654-65.
